# COVID-19 Is Not the Flu: Four Graphs From Four Countries

**DOI:** 10.3389/fpubh.2021.628479

**Published:** 2021-03-10

**Authors:** Jovana Stojanovic, Vincent G. Boucher, Jacqueline Boyle, Joanne Enticott, Kim L. Lavoie, Simon L. Bacon

**Affiliations:** ^1^Montreal Behavioral Medicine Centre, Centre integrée universitaire de santé et services sociaux du Nord de l'Ile de Montréal (CIUSSS-NIM), Montreal, QC, Canada; ^2^Department of Health, Kinesiology, and Applied Physiology, Concordia University, Montreal, QC, Canada; ^3^Department of Psychology, University of Quebec at Montreal, Montreal, QC, Canada; ^4^Monash Centre for Health Research and Implementation, Monash University, Clayton, VIC, Australia; ^5^School of Public Health and Preventive Medicine, Monash University, Melbourne, VIC, Australia; ^6^Monash Partners Academic Health Science Centre, Clayton, VIC, Australia

**Keywords:** COVID-19, influenza, transmission, epidemiology, behavior-based policy, behavior change

## Abstract

**Background:** COVID-19 has caused a global public health emergency. Government mitigation strategies included a series of behavior-based prevention policies that had a likely impact on the spread of other contagious respiratory illnesses, such as seasonal influenza. Our aim was to explore how 2019–2020 influenza tracked onto COVID-19 pandemic and its mitigation methods.

**Materials and Methods:** We linked the WHO FluNet database and COVID-19 confirmed cases (Johns Hopkins University) for four countries across the northern (Canada, the United States) and southern hemispheres (Australia, Brazil) for the period 2016–2020. Graphical presentations of longitudinal data were provided.

**Results:** There was a notable reduction in influenza cases for the 2019–2020 season. Northern hemisphere countries experienced a quicker ending to the 2019–2020 seasonal influenza cases (shortened by 4–7 weeks) and virtually no 2020 fall influenza season. Countries from the southern hemisphere experienced drastically low levels of seasonal influenza, with consistent trends that were approaching zero cases after the introduction of COVID-19 measures.

**Conclusions:** It is likely that the COVID-19 mitigation measures played a notable role in the marked decrease in influenza, with little to no influenza activity in both the northern and southern hemispheres. In spite of this reduction in influenza cases, there was still community spread of COVID-19, highlighting the contagiousness of SARS-CoV-2 compared to influenza. These results, together with the higher mortality rate from SARS-CoV-2 compared to influenza, highlight that COVID-19 is a far greater health threat than influenza.

## Introduction

On March 11th, 2020, the World Health Organization (WHO) declared COVID-19 a pandemic, a disease caused by the SARS-CoV-2 virus (1). As of December 31st, 2020, there have been around 83.52 million cases in 188 countries, areas, or territories, with a death toll of ~1.82 million individuals (2). COVID-19 prevention measures have relied upon widespread adherence to behavior-based policies, like physical distancing, mask wearing, and hand washing, to reduce virus transmission, even with the current introduction of vaccines in numerous countries (3–5). Theoretically, these mitigation measures should also have positive impacts on other transmissible infectious diseases such as the influenza virus.

Seasonal influenza A and B epidemics generally occur between November and April across the northern hemisphere, and between late May and October across the southern hemisphere (6), time periods which have overlapped with various COVID-19 waves in the countries in both hemispheres. As such, we aimed to explore how the epidemiological pattern of the 2019–2020 influenza tracked onto the evolution of the COVID-19 pandemic and the first introduction of behavior-based mitigation methods to prevent its transmission in four countries (Australia, Brazil, Canada, and the United States). Another aim was to provide evidence to combat the miss-information being spread (7) that the impact of COVID-19 is no different to influenza. These have propagated messages within some communities that adherence to the behavior-based prevention policies are unnecessary resulting in non-adherence and in some areas, public protesting against the policies (8).

## Materials and Methods

### Data Sources

We accessed FluNet data on influenza virological surveillance coordinated and provided by the WHO, between January 1st, 2016, and December 27th, 2020 (9). Influenza number of cases (A [H1]; A [H1N1] pdm09; A[H3]; A[H5]; A[not subtyped]; B [Yamagata lineage]; B [Victoria lineage]; B [lineage not determined]) and total number of influenza positive/negative viruses for Australia, Brazil, Canada, and the United States were downloaded for the period 2016–2020. Most recent COVID-19 epidemiological data, between January 1st and December 25th, 2020, were obtained from the Johns Hopkins Coronavirus Resources Center (2, 10). In order to obtain data on policies related to COVID-19, the Coronavirus Government Response Tracker was used to obtain the dates when the governments had first put in place closures and containment measures (school closing, workplace closing, international travel controls, canceling of public events) (11). For the northern hemisphere, the first containment measures included international travel controls on January 22nd and February 22nd for Canada and the United States, respectively. For the southern hemisphere, the international travel controls in Australia (February 2nd) and the school and workplace closing in Brazil (March 12th) were first introduced. Influenza and COVID-19 datasets were merged based on the weeks of the year (i.e., week 1 = the first week in January), and graphical presentations of the raw longitudinal data are provided in order to obtain instantaneous visual insight and discuss the potential influence of COVID-19 outbreak on influenza rates across the individual countries.

## Result

### Northern Hemisphere—Examples of Canada and the United States

**Figure 1:**
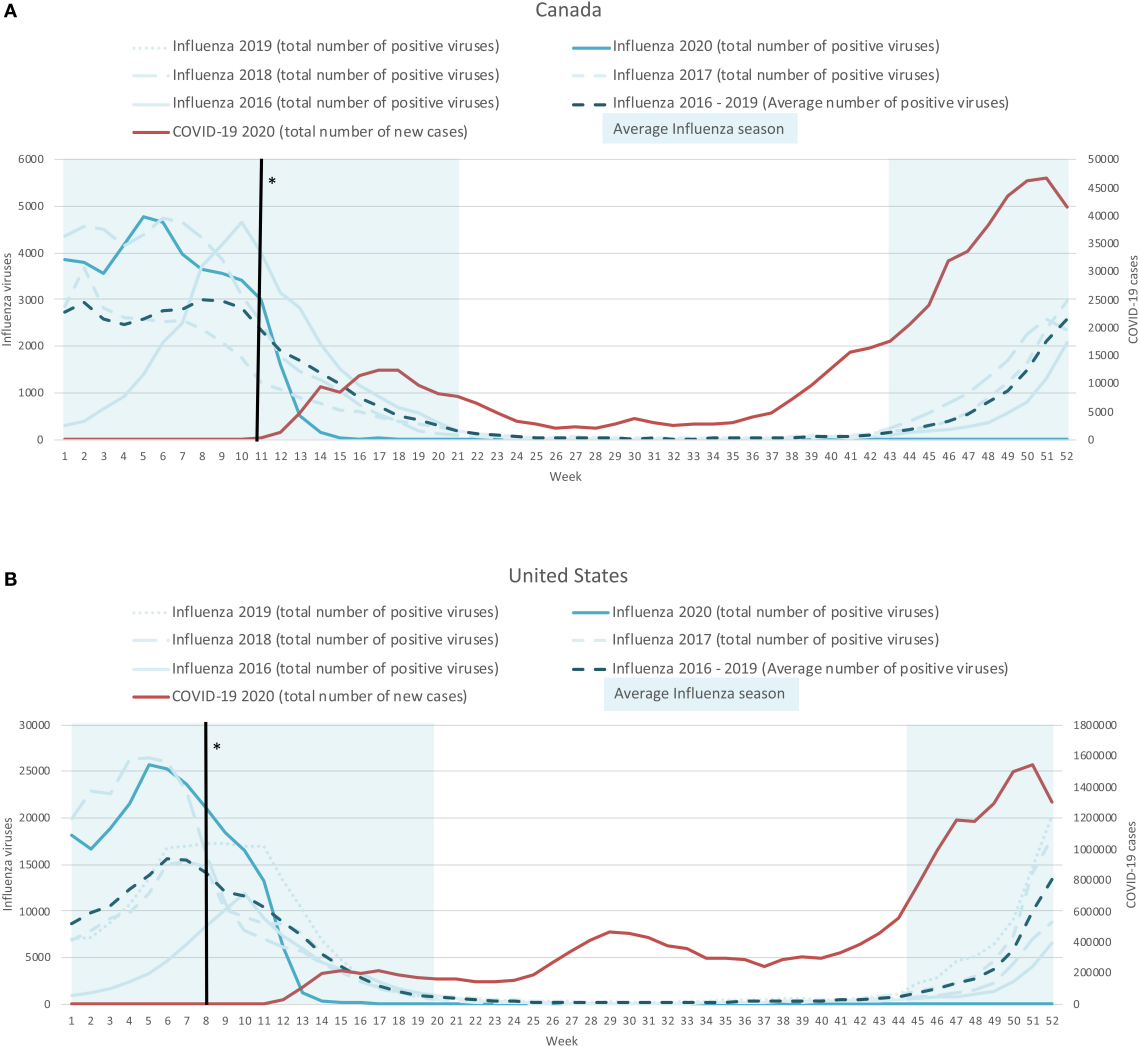
Weekly time-series presentation of COVID-19 cases (for 2020; red line) and influenza cases (for the seasons 2016–2020; blue lines) across the northern hemisphere [Case of Canada **(A)** and the United States **(B)**]. *The week in which the country implemented policy restrictions is noted with a black vertical line.

Between 2016–2019, the average influenza season occurred between October (week 40) and May (week 19) for both Canada and the United States, with peaks around weeks 7 and 8 (end of February). As seen in Figure 1, there was a significant reduction in influenza cases during the first months of 2020 (solid blue curve) compared to the average number of influenza cases (dashed dark blue curve) after the COVID-19 mitigation measures were introduced (solid black line). Furthermore, this notable decrease in influenza cases (lower than 500 cases/week) meant that the influenza season seemed to end 7 and 4 weeks earlier for the United States and Canada, respectively (week 15; between April 6 and 12, 2020) compared to previous years. In contrast, the number of cases of COVID-19 in both countries (solid red curve) started to increase dramatically during week 11 of 2020 (March 9–15, 2020), while there was a more rapid than usual decline in the number of influenza cases between weeks 12 and 13 (March 16–29, 2020). Finally, we observed persistently low numbers of influenza cases, approaching zero values, in both countries throughout the start of the 2020–2021 influenza season, which contrasts the consistent increases in cases and the second wave of COVID-19 (see Figures 1A,B).

### Southern Hemisphere- Examples of Australia and Brazil

**Figure 2:**
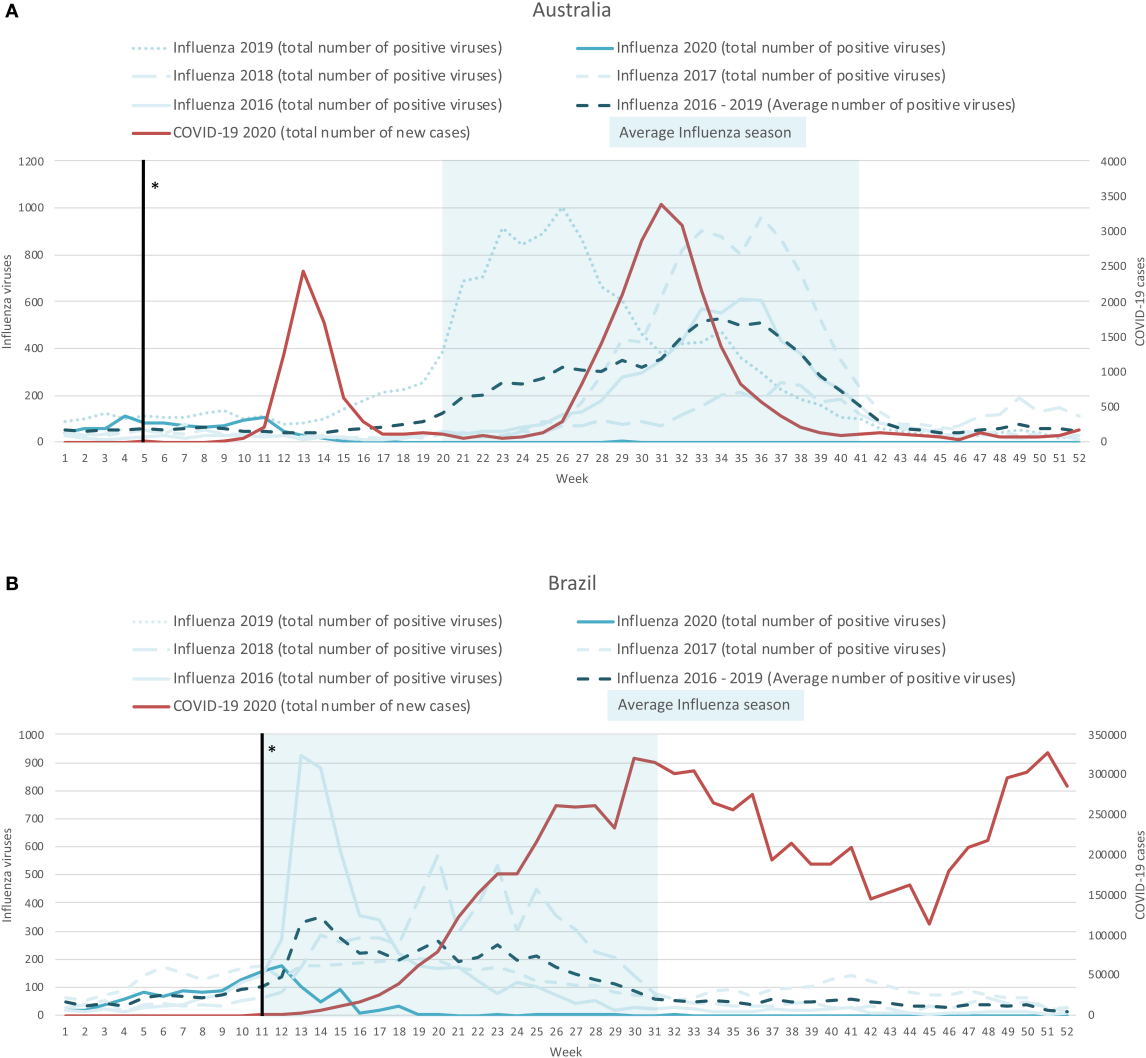
Weekly time-series presentation of COVID-19 cases (for 2020; red line) and influenza cases (for the seasons 2016–2020; blue lines) across the southern hemisphere [Case of Australia **(A)** and Brazil **(B)**]. *The week in which the country implemented policy restrictions is noted with a black vertical line.

In the southern hemisphere, the average influenza season runs from May (week 19) to November (week 45) in Australia and from March (week 9) to August (week 31) in Brazil, with peaks occurring around week 34 and week 14, respectively (see Figure 2). This pattern is notably different from the northern hemisphere. In Australia, the influenza case rates remained around zero from around week 16 up to when we stopped capturing data (December 27, 2020; week 40). More notable, is that the usual peak in influenza cases (around week 34) did not occur in 2020, this is in spite of there being a 2nd COVID-19 wave which covered this period (weeks 24–40, peak at week 31). In Brazil, the initial phases of the 2020 influenza season followed a normal pattern until week 12 (1 week after the introduction of the COVID-19 mitigation measures of the 4 countries included). By week 16 (mid-April, 2020) there were minimal influenza cases and by week 19 (early May, 2020) the case rate dropped to zero, where it remained until the end of data capture (December 27, 2020; week 40). In contrast, the number of COVID-19 cases started to increase around week 13, and have remained elevated ever since.

## Discussion

Our descriptive analysis of four countries across the southern and the northern hemispheres provides compelling evidence of the potential association between the waves of the COVID-19 pandemic, the introduction of behavior-based COVID-19 mitigation measures, and a reduction in influenza transmission. There are two notable implications stemming from our visual analysis, including: (1) the COVID-19 behavioral mitigation measures appear to be having an unintended positive consequence on influenza spread; and (2) the fact that there were still COVID-19 cases after the introduction of mitigation measures, and in the absence of community spread of influenza, suggests that these viruses are not equally transmissible.

With regards to the first point, even though our analyses are descriptive in nature, our hypothesis that the introduction of government measures, such as the adoption of widespread behavioral changes worldwide with respect to isolation, hygiene and social distancing in response to COVID-19 would have reduced the trends in annual influenza cases, remains plausible (12–14). We observed consistent reductions in influenza activity across the globe, which was most notable once governments had introduced their first measures. It is also worth highlighting that the four countries introduced their measures at different times in the year, yet their impact on the incidence of influenza cases was comparably rapid. Once the world has overcome the current COVID-19 pandemic crisis, consideration of the potential role of more rigorous and widespread implementation of the COVID-19 behavior-based prevention measures to curb the transmission of the influenza virus, and its global mortality burden (15), is needed.

To further emphasize the role of the behavior-based measures is the fact that most countries, including the United States and Canada, did not run a flu vaccine campaign for 2020, yet still managed to negate the usual incidence of influenza cases. This contrasts with Australia who ran an enhanced 2020 influenza vaccination campaign. When considering the impending distribution of the various COVID-19 vaccines, it might be possible for countries to drawn inspiration from this highly successful campaign, especially given the alarming increases in COVID-19 vaccine hesitancy (16). Elements of the Australian campaign included: continuous vaccination offer; aggressive public health messaging to inform the citizens, encourage vaccination and highlight the “*it's never too late to get the vaccine*” approach; increased advocacy for preventive behaviors; targeted messaging among at risk populations and healthcare workers; continuous surveillance and active monitoring (17). Importantly, it would seem that the development of public health policies and communication strategies aimed at increasing vaccine uptake might benefit from consultation with behavioral scientists, especially as the act of getting vaccinated is an important health behavior, whose insights have proven invaluable in the context of reducing COVID-19 transmission (18, 19).

Regarding our second implication, even though many of the parallels drawn between COVID-19 and influenza have already been discredited (20–26), it is often difficult to communicate this to the wider population. Our epidemiological mapping provides visually intuitive support to this difference which can help combat the misinformation that the impact of COVID-19 is no different to influenza. The current data clearly show how the two differ with respect to their infectiousness. The fact that there continues to be increases in COVID-19 cases with concomitant reductions in influenza spread around the globe highlights how viral the SARS-CoV-2 virus is.

## Limitations

Our study should be interpreted in consideration of some limitations. Firstly, our methodological approach included a simple graphical visualization strategy of raw data, and so causality cannot strictly be inferred from this descriptive analysis. Extending this, there could be some case misclassification with some COVID-19 cases actually being influenza cases. However, it should be noted that the data that we used was generally based on actual testing of both COVID-19 and influenza rather than just symptom reporting. Another limitation involves the nature of the recent influenza surveillance data. Case declines observed might be due to decreased testing over the course of the pandemic, as well as limited capacity for reporting in certain countries. This might be especially pertinent to low and middle-income countries where there is a historical lack of reliable estimates for influenza surveillance data. However, in the four countries highlighted in this paper this possible limitation might only apply to Brazil.

## Conclusion

Our report provides descriptive evidence that the behavior-based COVID-19 mitigation measures are likely to be associated with an important reduction in the transmission and impact of influenza. In spite of this reduction in influenza, there was still community spread of COVID-19, highlighting that SARS-CoV-2 is markedly more contagious compared to influenza. These graphs, together with the higher global mortality rates of SARS-CoV-2 compared to influenza, provide clear evidence that the impact of COVID-19 is far greater than influenza. Finally, greater implementation of some of the key behavior-based COVID-19 mitigation measures (18, 19, 27) to reduce the mortality and burden of future influenza outbreaks should be considered by governments.

## Data Availability Statement

The data presented in this study come from publically available online repositories. The names of the repository/repositories and accession links can be found at: FluNet (www.who.int/flunet), Global Influenza Surveillance and Response System (GISRS) (available at: https://apps.who.int/flumart/Default?ReportNo=2); The Oxford COVID-19 Government Response Tracker (OxCGRT) (available at: https://github.com/OxCGRT/covid-policy-tracker/tree/master/data); Center for Systems Science and Engineering at Johns Hopkins University. Coronavirus COVID-19 (2019-nCoV) Dashboard (available at: https://github.com/CSSEGISandData/COVID-19).

## Author Contributions

JS: conception and design, data analysis, interpretation of data, drafting the article, article reviewing and critical revision. VB: conception and design, data acquisition and analysis, interpretation of data, drafting the article, article reviewing and critical revision. JB, JE, and KL: conception and design, interpretation of data, article reviewing and critical revision. SB: conception and design, interpretation of data, drafting the article, article reviewing and critical revision.

## Conflict of Interest

SB has received consultancy fees from Merck for the development of behavior change continuing education modules, speaker fees from Novartis and Janssen, and has served on advisory boards for Bayer, Sanofi, and Sojecci Inc none of which are related to the current article. KL has served on the advisory board for Schering-Plow, Takeda, AbbVie, Almirall, Janssen, GSK, Boehringer Ingelheim (BI), and Sojecci Inc, and received sponsorship for investigator-generated research grants from GlaxoSmithKline (GSK) and AbbVie, speaker fees from GSK, Astra-Zeneca, Astellas, Novartis, Takeda, AbbVie, Merck, Boehringer Ingelheim, Bayer, Pfizer and Air Liquide, and support for educational materials from Merck, none of which are related to the current article. The remaining authors declare that the research was conducted in the absence of any commercial or financial relationships that could be construed as a potential conflict of interest.
